# Antimicrobial stewardship programs and antibiotic use in Africa: A systematic review and meta-analysis protocol

**DOI:** 10.1371/journal.pone.0334758

**Published:** 2025-10-17

**Authors:** Ammas Siraj Mohammed, Dawit Abrham

**Affiliations:** Clinical Pharmacy Department, School of Pharmacy, College of Health and Medical Sciences, Haramaya University, Harar, Ethiopia; The University of Lahore, PAKISTAN

## Abstract

**Background:**

Antimicrobial resistance (AMR) is a significant public health crisis, particularly in Africa where infectious disease burdens are high. While antimicrobial stewardship programs (ASPs) are a key intervention, a comprehensive synthesis of their impact on antibiotic use in this context is lacking.

**Objective:**

This systematic review and meta-analysis aims to synthesize current evidence regarding the association between ASPs and antibiotic use in Africa.

**Methods:**

MEDLINE (PubMed), Embase, CINAHL, Web of Science, and regional databases including african journals online and WHO global index medicus will be searched systematically. Original studies of the association of ASPs with antimicrobial consumption across healthcare settings will be included while Animal and environmental studies will be excluded. Two independent reviewers will screen articles, extract data, and assess quality using the Effective Public Health Practice Project (EPHPP) quality assessment tool. The main outcome measures will be proportion of patients receiving anantibiotic prescription and defined daily doses per 100 patient days. The pooled association of targeted ASPs with antimicrobial consumption will be be measured using multilevel random-effects models.

**Expected outcomes:**

This systematic review and meta-analysis is expected to provide valuable, pooled evidence on the effectiveness of ASPs in reducing antimicrobial consumption in Africa. The findings will inform and call for evidence based interventions by governments and Non-Governmental Organizations (NGOs) to combat AMR in resource limited settings.

**Systematic review registration:**

PROSPERO CRD420251003018.

## Introduction

Antimicrobial resistance (AMR) is a growing global health crisis, posing a significant threat to the effective treatment of infectious diseases [1]. Globally, 4.95 million deaths were associated with bacterial AMR in 2019, with the greatest number of deaths per head of population in Africa [2]. The African continent faces a particularly acute challenge due to a convergence of factors, including high rates of infectious diseases, limited access to diagnostic facilities, and widespread inappropriate antibiotic use [3]. This situation contributes to the rapid emergence and dissemination of AMR, jeopardizing public health outcomes [3,4]. A recent Africa CDC report warns that AMR is a more significant threat to the continent than HIV/AIDS, tuberculosis, and malaria combined [5].

The inappropriate use of antibiotics, driven by factors such as over-prescription, self-medication, and inadequate infection control practices, is a major driver of AMR. In Africa, these issues are exacerbated by weak healthcare systems, limited resources, and a lack of robust surveillance mechanisms [6]. This context stands in stark contrast to high-income countries, where ASPs are a standard of care and a cornerstone of patient safety. Antimicrobial stewardship programs are recognized as a crucial strategy to optimize antibiotic use and mitigate the spread of AMR [7]. These programs aim to promote the rational use of antibiotics through various interventions, including education, guidelines, and monitoring [8]. A global study has depicted implementing ASPs was associated with a 10% decrease in antibiotic prescriptions and 28% reduction in antibiotic consumption [9].

Additionally, a systematic review and meta-analysis study conducted in USA suggests that ASPs can reduce usage of restricted antimicrobial medications by 27% and overall antibiotic consumption by 19% in hospital [10].

The global evidence base for ASPs is notably dominated by studies from high-income countries, which have different healthcare systems and antibiotic use patterns [11]. Furthermore, existing research suggests that effectiveness ASPs in different settings can be different due to variable infrastructure, resources, and cultural beliefs [12,13]. There is also evidence that tailored ASPs can lead to significant improvements in antibiotic prescribing practices and reductions in AMR [13,14].

While the importance of ASPs is well-established, their implementation and evidence of effectiveness in the African context remain variable [15]. There is currently a dearth of comprehensive data regarding the efficacy of ASPs in Africa, where the usage of antibiotics is remarkably elevated in comparison to high income countries (HICs) [16,17].

Therefore, a comprehensive synthesis of the available evidence is needed to assess the impact of ASPs on antibiotic use in Africa. This systematic review and meta-analysis protocol aims to quantify the effect of ASPs on antibiotic consumption and prescribing patterns, identify factors that influence their effectiveness in the region, and provide evidence-based recommendations for their context-effective implementation. Based on preliminary searches of major databases and systematic review registries, we have found no published protocol or completed review that addresses this critical research gap.

### Study objectives

#### Primary objective.

The primary objective of this systematic review and meta-analysis is to determine the pooled effect of ASPs on antibiotic use.

#### Secondary objectives.

The secondary objectives of this study are to examine antibiotics prescribing patterns, factors influencing effectiveness, and types of ASP interventions.

### Review questions

The question of this review include:

What is the impact of ASPs on antibiotic and prescribing patterns in African healthcare settings?

What is the effectiveness of different ASP interventions on antibiotic use in Africa?

What are the factors that influence the effectiveness of ASPs in this African region?

### Inclusion criteria

**Participants:** All Patients, including pediatrics and adults, receiving antibiotic treatment in African healthcare facilities and healthcare professionals involved in antibiotic prescribing and administration in African healthcare facilities and healthcare facilities themselves, as the units where ASPs are implemented will be participants of this study.

**Interventions:** This systematic review and meta-analyses will include RCTs, quasi-experimental, and observational studies that examines ASPs in Africa. Antimicrobial stewardship programs in this context include educational programs for healthcare professionals, implementation of antibiotic usage guidelines, audit and feedback mechanisms and Restriction of certain antibiotics.

**Comparisons:** Usual care or no Antimicrobial stewardship programs.

**Outcomes:** This review will consider studies that include the following outcomes; Primary outcome is defined daily dose (DDD) per 100 or 1000 patient days (PDs) or days of therapy (DOT) per 100 or 1000 PDs. The percentage of patients who receive an antibiotic prescription will be the the secondary outcome of this study. Additionally, adherence to guidelines, resistance rate and length of stay will be determined where reported.

### Phenomena of interest

This review will include original studies conducted to assess the impact of antimicrobial stewardship programs on antibiotic use across Healthcare settings, including hospitals and community settings in Africa.

### Context

Original articles conducted in Healthcare settings in Africa will be included in the systematic review and meta-analyses.

### Types of research syntheses

This systematic review and meta-analyses will consider original studies of all different designs. The goal of EPHPP systematic review and meta-analyses is to reveal pooled magnitude of effectiveness [18,19]. The review will also include qualitative syntheses of research articles.

## Methods

The planned review of systematic review and meta-analyses will be carried out using the EPHPP systematic review and meta-analyses methodology [18]. The title for this review was registered on PROSPERO and obtained registration ID CRD420251003018.

### Search strategy

Comprehensive search including both published and unpublished research syntheses as per JBI Methodological recommendation [20,21] will be sought after using the search strategies. In this review, a three-step search methodology will be used. First, an initial limited search of PubMed (S1 File) and CINAHL (EBSCO) will be undertaken to identify articles on the topic. The text words contained in the titles and abstracts of relevant articles, and the index terms used to describe the articles will be used to develop a full search strategy for reporting the name of the relevant databases/information sources. The search strategy, including all identified keywords and index terms, will be adapted for each included database and/or information source. The reference list of all included sources of evidence will be screened for additional studies. Studies published in any language will be considered for inclusion. Non-English articles will be translated using bilingual collaborators and appropriate translation tools.

Original articles conducted in Africa on the impact of antimicrobial stewardship programs on antibiotic use across healthcare settings irrespective of year of publication fulfilling inclusion criteria will be included.

### Information sources

The databases will be searched for this review include MEDLINE (PubMed), Embase, CINAHL and Web of Science, as well as regional and multilingual databases such as African Journals Online (English, French, Portuguese), and WHO Global Index Medicus (AIM, LILACS, IMEMR). To capture unpublished reviews, additional searches will be conducted using Google Scholar and institutional websites such as WHO website, CDC website, and National Ministries of Health websites of African countries.

### Study selection

The studies retrieved through database searching and other sources will be screened for relevance, and those identified as being potentially eligible will be fully assessed against the inclusion/exclusion criteria, and selected or rejected, as appropriate (S2 File). The duplicate will be removed by using the Endnote software. Critical appraisal will be conducted to assess the internal (systematic error level) and external validity (generalizability) of studies, and to reduce the risks of biases. The mean scores of two authors will be taken into account in coming to a final decision regarding study quality, and studies with scores greater than or equal to half the required points will be included in the data analysis. A third author will resolve any discrepancies arising between the two authors during the assessment process. The quality of the included studies will be evaluated by two reviewers independently using the Joanna Briggs Institute Critical Appraisal Tools (for observational studies).

### Assessment of methodological quality

Selected syntheses will be critically appraised by two independent reviewers (AS and DA) for methodological quality in the review using Effective Public Health Practice Project (EPHPP) quality assessment tool to assess 6 domains of quality: (1) selection bias, (2) design, (3) confounders, (4) blinding, (5) data collection methods, and (6) withdrawal and dropouts. EPHPP is a widely used assessment tool for quantitative studies designed for systematic literature reviews of effectiveness studies. [18]. Authors of papers will be contacted to request missing or additional data for clarification, where required. Any disagreements that arise between the reviewers will be resolved through discussion, or with a third reviewer. The results of the critical appraisal will be reported in narrative form and in a table. All syntheses, regardless of the results of their methodological quality, will undergo data extraction and synthesis.

### Data collection

Quantitative data will then be extracted from the papers selected for inclusion using the standardized JBI data extraction tool. The authors will extract important data related to the study characteristics (the region and the study area, the first author, the year of publication, the study design, the population characteristics, and the sample size) and the outcomes of interest (the effect size data, including the antimicrobial stewardship program, and the antibiotic use). Any disagreements that arose between the reviewers were resolved through discussion or with a third reviewer. Authors of papers were contacted to request missing or additional data, where required. Any papers with missed information after two contacts of authors of the included studies will be excluded if no response obtained at after the second contact.

### Data summary

Data from included studies will be extracted using tool prepared in Microsoft Excel and exported to Stata version 18.0 for analyses of the pooled estimates of primary and secondary outcome measures, as well as for subgroup analysis. Considering variation in true effect sizes across the population (clinical heterogeneity), DerSimonian and Laird’s random effects model will be applied for the analyses, with a 95% confidence level and interpret heterogeneity using the I² statistic with defined thresholds (I² < 30%, low; I² = 30–60%, moderate; I² > 60%, High heterogeneity). Accordingly, the event size (proportion) will be calculated, and standard error of Logit event rate will be determined using Stata software. A quantitative synthesis will be carried out using random effects model. We will also provide a narrative synthesis of the findings, structured around the predictive factors investigated, the target population characteristics, and the type of outcome content. Summaries of the strength of the association between the prognostic risk factors and the outcomes for each study will also be provided by calculating risk ratios (for dichotomous outcomes) or standardised mean differences (for continuous outcomes). The presence of publication bias will be evaluated using the Begg and Mazumdar rank correlation test, as well as Egger’s regression test, and presented using funnel plots. Subgroup analyses will be carried out for the different regions of Africa.

### Assessing certainty in the outcome

The Grading of Recommendations, Assessment, Development and Evaluation (GRADE) approach for grading the certainty of evidence will be followed and a Summary of Findings (SoF) will be created using GRADE pro GDT [22]. We will use the GRADE approach to determine how confident we are in the evidence from the reviews we include. The GRADE approach evaluates four aspects: the quality and design of the original studies, how consistent their results are, and how relevant they are to our question.

We will rate the evidence as high, moderate, low, or very low quality. We will also summarize the findings for the four outcomes that our review focuses on: Antibiotics consumption, prescribing practice, ASPs effectiveness, and broad spectrum antibiotics prescribing [21]. This will be undertaken by two independent reviewers (AS and DA) at the outcome level. Any disagreements that arise between the reviewers will be resolved through discussion or with a third reviewer.

Authors of papers were contacted to request missing or additional data for clarification, where required through two emails request. The SoF will present the following information where appropriate: absolute risks for the treatment and control, estimates of relative risk, and a ranking of the quality of the evidence based on the risk of bias, directness, heterogeneity, precision and risk of publication bias of the review results. Two different kinds of outcome measurements are used in the current literatures [23]. First, defined daily dose (DDD) per 100 or 1000 patient days (PDs) or days of therapy (DOT) per 100 or 1000 PDs are the two common ways to quantify real drug consumption. Drugs administered as multiple doses are measured by DDD, anticipated daily maintenance dose for a particular patient, usually an adult. Regardless of the number of doses or dosage strength, DOT is the number of days of antibiotic therapy given to a patient [18]. We will use mixed DDD and DOT in the meta-analysis because they have conceptual similarities. All DDD and DOT measurements will be normalized to 100 PDs. The percentage of patients who receive an antibiotic prescription will be the secondary outcome; this is a different metric that does not directly assess drug usage. We will calculated one of two outcomes for each study: (1) the difference in antibiotic prescriptions between before and after the intervention, or (2) the rate ratio (RR) of antibiotic consumption between the pre-intervention and post-intervention periods, expressed in DDD or DOT per 100 PDs. Additionally, adherence to guidelines, resistance rate and length of stay will be determined where reported.

### Status and timeline of the study

The review began in July 2025, and the official literature search will be finished by the end of September 2025. Results will be presented in three primary formats: summary tables, visual figures, and a systematic synthesis. A PRISMA flow diagram will be presented for the study selection process. A concept map will be developed to illustrate the relationships among the main themes identified in the review, providing an overview of the findings. The dissemination strategy will involve publishing the review results in a peer-reviewed, open- access healthcare journal and presenting the findings at prestigious scientific conferences. Findings will be disseminated to an academic audience by February 2026.

## Discussion

Antimicrobial resistance (AMR) is a global health threat closely linked to the inappropriate use of antibiotics, which can diminish the effectiveness of antibiotics. It is a significant and growing problem in Africa, with high rates of antibiotic resistance reported. Inappropriate antibiotic use is a major driver of AMR, making ASPs crucial. Many African countries face resource limitations, which can hinder the implementation and sustainability of ASPs. Lack of trained personnel, limited laboratory capacity, and inadequate funding are common challenges. The prevalence and effectiveness of ASPs vary significantly across African countries due to differences in healthcare systems, resources, and policies. ASPs have the potential to significantly reduce inappropriate antibiotic use in Africa. Strategies such as antibiotic formulary restrictions, pre-authorization requirements, and prospective audit and feedback can promote rational antibiotic prescribing.

Despite the potential benefits, ASP implementation faces challenges, including: Lack of awareness and understanding of ASP principles, Resistance from healthcare providers, Weak healthcare infrastructure. Effective ASPs in Africa require context-specific approaches that consider local epidemiology, resource availability, and cultural factors. The WHO, and various other organizations are pushing for the implementation of ASP’s across the African continent. There is a need for more research to evaluate the effectiveness of ASPs in different African settings. Improved surveillance of antibiotic use and resistance is essential to monitor the impact of ASPs. While ASPs are recognized as a vital tool in combating AMR in Africa, their successful implementation requires addressing significant challenges. Efforts are being made to promote ASPs across the continent, but sustained commitment and investment are needed.

As far as researchers’ knowledge is concerned this systematic review and meta-analyses is of its first kind in Africa on ASPs and antibiotics use. The review will focus to identify the overall pooled magnitude of antibiotics consumption, and pooled association of targeted ASPs with antimicrobial consumption in Africa. The result will helps to provide evidence-based recommendations for the implementation of effective ASPs in Africa, which might be base to design policies and strategies to fight against anti-microbial and irrational use of antibiotics across different healthcare settings in Africa.

All published and unpublished original article in Africa setting will be considered better estimating the overall pooled magnitude of antibiotics consumption, and pooled association of targeted ASPs with antimicrobial consumption in Africa. Hence, this systematic review and meta-analyses is required to reveal the overall pooled magnitude of antibiotics consumption, and pooled association of targeted ASPs with antimicrobial consumption in Africa. It is also essential opportunity with study to state the status of ASPs and its impacts on antibiotics use in Africa. The study will also significantly important to embrace the factors that influence the association between ASPs and antibiotic use in Africa, to enable tailored intervention will be designed for each factor.

The result from this review will attract the attentions of government and partners for ASPs which is serving as driving engine to fight against anti-microbial resistance. Furthermore, it serves for policy makers by providing evidence on the impacts of ASPs on antimicrobial use to design context specific intervention to optimize antimicrobial use and halt misuse of antimicrobial. Parallel to this, it will supplement evidence for government to develop guidelines, policies and proclamations for use of antimicrobial use which will play pivotal role in preventing anti-microbial resistance. The result form this review will essential for appropriate implementation of across all healthcare settings in Africa. It can be taken as a benchmark for future researchers undertaking their interventional and other high standard studies to optimize ASPs and reduce anti-microbial resistances.

All efforts will be made to disseminate the evidence to scientific community through different means like present the work on different workshops, conference and publication on internationally reputable scientific journal. However, the might be limitation of the review involves potential for publication bias, the heterogeneity of ASP definitions across studies, and vthe finding of this review should use with caution to the other areas than within Africa.

## Conclusion

The finding will used as a basic information for understanding the impact of ASP on antibiotics use in Africa. Furthermore, this review will aid for design of context specific tool to implement ASP in Africa as antimicrobial resistance is one of the global concerns currently. In addition, the result will explore importance of tailored ASP implementation in prevention of anti-microbial resistance- Global concerns of our time. Provide evidence-based recommendations for the implementation of effective ASPs in Africa.

## Supporting information

S1 File This is a complete draft of our search strategy for the PubMed database. (DOCX)

S2 File This is the preferred reporting items for systematic review and meta-analysis protocols (PRISMA-P) 2015 statement. *Systematic Reviews* 2015.
(DOCX)
