# Supplementary material for: Antimicrobial stewardship programs and antibiotic use in Africa: A systematic review and meta-analysis protocol
Source: PLoS One. 2025 Oct 17;20(10):e0334758. doi: 10.1371/journal.pone.0334758 (PMC12533884; doi:10.1371/journal.pone.0334758)
Supplement: S1 File — (DOCX) [file pone.0334758.s001.docx]

**Supplementary 1**

**Complete draft of our search strategy for the PubMed database**

The literature search will be conducted using keywords related to Antimicrobial stewardship programs and antibiotics use in Africa (Antimicrobial stewardship (MeSH) OR “antibiotics stewardship” OR “ASP” OR “Antibiotic control” OR “antimicrobial management” AND (‘‘antibiotic use’’ OR ‘‘antibiotics consumption’’ OR ‘‘antimicrobial use’’) AND Africa ((“Algeria’’, ‘‘Angola”, “Benin”, “Botswana”, “Burkina Faso”, “Burundi”, “Cameroon”, “Cape Verde”, “Central African Republic”, “Chad”, “Comoros”, “Congo”, “Côte d'Ivoire”, “Djibouti”, ‘‘ Egypt’’, “Equatorial Guinea”, “Eritrea”, “Ethiopia”, “Gabon”, “Gambia”, “Ghana”, “Guinea”, “Guinea-Bissau”, “Kenya”, “Lesotho”, “Liberia”, ‘‘ Libya’’, “Madagascar”, “Malawi”, “Mali”, “Mauritania”, ‘‘ Morocco’’, “Mozambique”, “Namibia”, “Niger”, “Nigeria”, “Rwanda”, “Sao Tome and Principe”, “Senegal”, “Sierra Leone”, “Somalia”, “South Africa”, “Sudan”, “Swaziland”, “Tanzania”, “Togo”, ‘‘Tunisia’’, “Uganda”, “Zambia”, and “Zimbabwe”) AND (‘‘Healthcare settings’’ OR ‘‘Hospitals’’). The Boolean operators (AND, OR, NOT) will be used to refine the searching, and truncation will be applied in order to include plural and singular results. The search term will be connected to each other through ‘‘AND” or ‘‘OR” and by ‘‘OR” to their MeSH term. Published and unpublished articles available online up to the day of data collection will be considered for inclusion.
